# Antibiotic impregnated catheters and intrathecal antibiotics for CSF shunt infection prevention in children undergoing low-risk CSF shunt surgery

**DOI:** 10.1186/s12887-024-04798-9

**Published:** 2024-05-11

**Authors:** Stacey Podkovik, Chuan Zhou, Susan E. Coffin, Matthew Hall, Jason S. Hauptman, Matthew P. Kronman, Francesco T. Mangano, Ian F. Pollack, Sabrina Sedano, Joaquin Vega, Joshua K. Schaffzin, Emily Thorell, Benjamin C. Warf, Kathryn B. Whitlock, Tamara D. Simon

**Affiliations:** 1 Department of Neurological Surgery, Riverside University Health Sciences Medical Center, Riverside, CA USA; 2 Center for Child Health, Seattle Children’s Research Institute, Behavior, and Development, Seattle, WA USA; 3 Department of Pediatrics, School of Medicine, University of Washington, Seattle, WA USA; 4 Department of Pediatrics, University of Pennsylvania School of Medicine, Philadelphia, PA USA; 5 Children’s Hospital Association, Lenexa, KS USA; 6 Department of Neurological Surgery, University of Washington School of Medicine, Seattle, WA USA; 7 Department of Neurosurgery, University of Cincinnati College of Medicine, Cincinnati, OH USA; 8 Department of Neurological Surgery, University of Pittsburgh School of Medicine, Pittsburgh, PA USA; 9 Division of Hospital Medicine, Children’s Hospital Los Angeles, 4650 Sunset Blvd,, MS 94, Los Angeles, CA 90027 USA; 10 Faculty of Medicine, University of Ottawa, Ottawa, ON Canada; 11 Department of Pediatrics, University of Utah School of Medicine, Salt Lake City, UT USA; 12 Department of Neurosurgery, Harvard School of Medicine, Boston, MA USA; 13 New Harmony Statistical Consulting, Clinton, WA USA; 14 Department of Pediatrics, University of Southern California Keck School of Medicine, Los Angeles, CA USA

**Keywords:** CSF, Hydrocephalus, Infection, Antibiotic impregnated catheter

## Abstract

**Background:**

Cerebrospinal fluid (CSF) shunts allow children with hydrocephalus to survive and avoid brain injury (J Neurosurg 107:345-57, 2007; Childs Nerv Syst 12:192-9, 1996). The Hydrocephalus Clinical Research Network implemented non-randomized quality improvement protocols that were shown to decrease infection rates compared to pre-operative prophylactic intravenous antibiotics alone (standard care): initially with intrathecal (IT) antibiotics between 2007–2009 (J Neurosurg Pediatr 8:22-9, 2011), followed by antibiotic impregnated catheters (AIC) in 2012–2013 (J Neurosurg Pediatr 17:391-6, 2016). No large scale studies have compared infection prevention between the techniques in children. Our objectives were to compare the risk of infection following the use of IT antibiotics, AIC, and standard care during low-risk CSF shunt surgery (*i.e.,* initial CSF shunt placement and revisions) in children.

**Methods:**

A retrospective observational cohort study at 6 tertiary care children’s hospitals was conducted using Pediatric Health Information System + (PHIS +) data augmented with manual chart review. The study population included children ≤ 18 years who underwent initial shunt placement between 01/2007 and 12/2012. Infection and subsequent CSF shunt surgery data were collected through 12/2015. Propensity score adjustment for regression analysis was developed based on site, procedure type, and year; surgeon was treated as a random effect.

**Results:**

A total of 1723 children underwent initial shunt placement between 2007–2012, with 1371 subsequent shunt revisions and 138 shunt infections. Propensity adjusted regression demonstrated no statistically significant difference in odds of shunt infection between IT antibiotics (OR 1.22, 95% CI 0.82–1.81, *p* = 0.3) and AICs (OR 0.91, 95% CI 0.56–1.49, *p* = 0.7) compared to standard care.

**Conclusion:**

In a large, observational multicenter cohort, IT antibiotics and AICs do not confer a statistically significant risk reduction compared to standard care for pediatric patients undergoing low-risk (*i.e.,* initial or revision) shunt surgeries.

## Introduction

Cerebrospinal fluid (CSF) shunts allow children with hydrocephalus, a common cause of neurological disability [1, 2], to survive and avoid ongoing brain injury. A recent meta-analysis demonstrated a pooled incidence of congenital hydrocephalus in the United States at 68 per 100,000 live births [3], with nearly 400,000 new cases of hydrocephalus globally per year [3, 4] CSF shunt placement has been the mainstay of hydrocephalus treatment for over 60 years [5]. However, CSF shunts are associated with repeated revision surgeries and risk of infection [5]. Mechanical malfunction is frequent, and 60% of shunts require surgical revision within 4 years [6–8]. In the United States there are approximately 20,000 pediatric CSF shunt surgeries annually [9]. With each CSF shunt surgery, the risk of shunt infection increases [10, 11], and in the United States there are approximately 2,000 pediatric CSF shunt infections per year [9]. The burden to children, families, and the healthcare system of CSF shunt infections in terms of costs [9], morbidity over the life span [12], and quality of life [13] are substantial and preventable [14].

Controversies have emerged in the field of hydrocephalus about optimal peri-operative techniques to prevent CSF shunt infections in addition to the now-standard use of prophylactic IV antibiotics [11]. The BASICS trial demonstrated that antibiotic impregnated catheters (AIC) had lower infection rates as compared to standard shunt catheters, while silver-impregnated catheters did not, in a mixed population of children and adults in the United Kingdom [15]. The Hydrocephalus Clinical Research Network (HCRN) has instituted multiple quality improvement protocols that have been shown to decrease infection rates compared to pre-operative prophylactic antibiotic administration: initially with intrathecal (IT) antibiotics between 2007–2009 [16], followed by its replacement with AIC in 2012–2013 in North America [17]. Despite the benefit of AICs suggested in the BASICS trial, there have been few large scale studies directly comparing different infection prevention techniques that have shown to be superior to standard shunt catheters in children and none in a low-risk population [18, 19]. The aim of this study was to compare the odds of infection following the use of IT antibiotics, AIC, and standard care during low-risk CSF shunt surgery in children using large scale, multi-center administrative data augmented with clinical data.

## Design/methods

### Study design and setting

This was a retrospective cohort study conducted at 6 large pediatric neurosurgical practices at tertiary care children’s hospitals (Boston Children’s Hospital, Children’s Hospital of Philadelphia, Children’s Hospital of Pittsburgh, Cincinnati Children’s Hospital Medical Center, Primary Children’s Hospital, and Seattle Children’s Hospital) between 2007 and 2015 [20]. These hospitals were selected due to their inclusion in the Pediatric Health Information System + (PHIS + ; Children’s Hospital Association, Lenexa, KS) database that includes detailed administrative, laboratory, microbiology, and radiology data for all children receiving care at participating centers [20]. The hospital names were blinded for the presentation of the results; Hospitals A, D, E and F were all HCRN sites [20].

### Study population

The study population included children ≤ 18 years of age who underwent initial shunt placement between January 1, 2007 and December 31, 2012 at one of the six study sites [20]. During the screening phase, medical records for 5,903 children and 11,121 shunt procedures were abstracted from PHIS based on evidence of initial shunt placement or shunt revision between January 1, 2007 and December 31, 2012. Initial CSF shunt placements were defined as admissions with any *International Classification of Diseases, Ninth Revision, Clinical Modification* procedure code for extracranial ventricular shunt placement (02.3–02.35 except 02.39 alone), excluding those with any concurrent procedure code for replacement (02.42) or removal of ventricular shunt (02.43), and/or any diagnosis code for shunt malfunction (996.2), and/or shunt infection (996.63) [20]. CSF shunt revisions were defined as admissions with a primary diagnosis code for shunt malfunction (996.2) excluding those with concurrent CSF shunt infection (996.63) [20]. Dates of initial shunt placement and any subsequent revision surgeries were also abstracted [20]. Medical records were screened by trained study staff at each participating site to confirm that each initial shunt placement identified through PHIS screening represented that child’s true initial placement and that we were able to capture details of any preceding neurosurgical procedures [20]. Surgical procedure data including all initial CSF shunt placements, CSF shunt revisions, and first CSF shunt infections was collected for each eligible child through December 31, 2015, allowing each child at least three years of follow up time since the initial shunt placement [20].

### Data sources

The PHIS + database was augmented with detailed clinical data obtained from chart review to create a database with over 3,000 CSF shunt surgeries for the investigation of CSF shunt infection prevention. This approach permitted us both to confirm critical variables (e.g. use of IT antibiotics and AIC) and to obtain additional variables unavailable in PHIS + (e.g. surgical decisions in the operating room).

All site investigators participated in a group consensus process to determine which additional variables were feasible and accurate to collect in chart review. We used Research Electronic Data Capture (REDCap), a secure web-based application for electronic data capture, to ensure consistent chart review data collection across sites [21, 22]. Data obtained through chart review were matched to PHIS + data using hospital, medical record number, and date of surgery.

A comprehensive data quality assurance plan, explained in detail in Podkovik et al*.*, was implemented to ensure that data collected from PHIS and PHIS + adhered to internally consistent definitions and accurately reflected clinical course and outcomes [20].

### Outcome variables

The outcome of CSF shunt infection was defined adopting the widely-used HCRN consensus definition of CSF shunt infection, which [11, 16, 17] is either 1) microbiological determination of presence of bacteria in culture of CSF, wound swab, and/or pseudocyst fluid; or 2) shunt erosion (visible hardware); or 3) abdominal pseudocyst (even without positive culture) [11, 16, 17]. The primary outcome was infection within 6 months from the most recent surgery. Subjects were censored at the time of their first infection or at the conclusion of the observation period, whichever came first.

Secondary outcomes included length of hospital stay (days) and rates of post-operative complications: bacteremia, CSF leak, pseudomeningocele, meningitis, need for antibiotic treatment for wound site, bowel perforation and other complications.

### Predictor variables

We took advantage of the natural experiment that occurred in PHIS + hospitals from 2007 to 2012 with the use of IT antibiotics and AIC. During the study period, most patients received standard care, defined as receiving prophylactic IV antibiotics (either cefazolin or vancomycin) without IT antibiotics and having conventional shunt tubing.

IT antibiotics were defined by an appropriate antibiotic (e.g., vancomycin, gentamicin) with an appropriate intrathecal dose (i.e., 0–10 mg). Corroborating information from the operative report and/or surgeon survey were likewise evaluated.

AIC use was determined by documentation from the operative report. Corroborating information from the operative report and/or surgeon survey were likewise evaluated.

All outcomes were associated with the technique used in the preceding CSF shunt surgery. Because a given patient may undergo multiple CSF shunt surgeries for which different infection prevention techniques might be used, the predictor variables are time-varying in the analysis.

### Statistical analysis

For descriptive statistics we reported means and standard deviations for the continuous variables. For categorical variables we reported counts, proportions and 95% confidence intervals.

Due to the observational nature of our study design, we performed propensity score analyses with inverse probability treatment weighting to estimate the relationship between prevention techniques (standard technique, intrathecal antibiotics, and antibiotic impregnated catheter) and shunt infection within 6 months of shunt placement. The propensity score, defined as the conditional probability of receiving treatment given covariates, plays a central role in causal inference. Under certain assumptions, an unbiased estimate of the average treatment effect can be obtained by adjusting for the propensity score alone rather than a vector of covariates, which is often of high dimension [23].

For our primary analysis, we first applied the covariate balancing propensity score (CBPS) methodology [24] to model the probability of shunt infection prevention techniques while optimizing the covariates balance [20]. The CBPS takes advantage of the dual characteristics of the propensity score as a covariate balancing score and the conditional probability of treatment assignment. This method does two things simultaneously: it 1) balances covariates and 2) optimizes predicted probability of treatment given covariates. CBPS has been extended to more than two treatment options. We estimated CBPS for initial shunt placement and revision placement separately. A list of predetermined covariates based on previous research [10, 11, 25–28] were included in the CBPS models: patient age, patient biological sex, patient race, primary insurance, patient weight, weekday or weekend of the procedure, complex chronic conditions, admission priority, etiology of patient hydrocephalus, concurrent non-neurosurgical procedure, concurrent neurosurgical procedure, prior CSF leak, prior gastrostomy, prior inpatient antibiotics, prior CNS surgeries, prior non-CNS surgeries, and prior tracheostomy. The CBPS and weights were calculated using [29] and [30] packages in R (R Core Team, 2022) [31]. Covariate balance between infection prevention techniques in the propensity score weighted sample was assessed by balance tables and density plots, using the {cobalt} [32] package in R. After we derived CBPS weights, inverse probability treatment weighting was applied to logistic regression models, with infection within 6 months as outcomes, and infection prevention techniques as the sole predictor. We reported adjusted odds ratios (aORs) and 95% confidence intervals. For our secondary analyses, continuous outcome variables were compared using a Kruskal–Wallis rank sum test, and binary outcomes were compared using Fisher’s Exact Test. All analyses were conducted in R statistical software (R Core Team, 2022) version 4.2.2.

### Role of the funding source

The content is solely the responsibility of the authors and does not necessarily represent the official views of the National Institutes of Health.

## Results

From a total of 5,903 unique patients within the PHIS + data set, 1,723 patients had an initial shunt placed amongst six PHIS + pediatric hospitals between January 1, 2007 and December 31, 2012. These children experienced 3,094 initial shunt placements and shunt revisions prior to development of first CSF shunt infection or censoring at the end of the observation period, December 31, 2015. Patient-level demographics at the time of the initial shunt placement are provided in Table 1.

**Table 1:** Patient-level characteristics for the overall cohort and in association with CSF shunt infection within 6 months

Total patients	*n* = 1,723	Infection present (*n* = 89)	Infection absent (*n* = 1,634)
**Gender**, *n* (%)			
Male	995 (58)	57 (64)	938 (57)
Female	728 (42)	32 (36)	696 (43)
**Race**, *n* (%)			
White	1266 (75)	67 (75)	1199 (75)
Black	208 (12)	15 (17)	193 (12)
Asian	27 (2)	2 (2)	25 (2)
Mixed	24 (1)	1 (1)	23 (1)
Other	151 (9)	4 (5)	147 (9)
**Ethnicity**, *n* (%)			
Hispanic	183 (13)	5 (6)	178 (13)
Non-Hispanic	1215 (87)	73 (94)	1142 (87)
**Birth history**			
**Birth weight**, grams, mean (SD)	2723 (1112)	2883 (1119)	2712 (1111)
**Gestational age**, months, median (IQR)	37 (33,39)	37 (33,39)	37 (33,39)
Hydrocephalus etiology^*^, *n* (%)			
CNS^a^ tumor	337 (20)	11 (12)	326 (20)
Myelomeningocele	283 (16)	14 (16)	269 (16)
IVH^b^	221 (13)	10 (11)	211 (13)
Congenital	133 (8)	15 (17)	118 (7)
CCH^c^	132 (8)	4 (5)	128 (8)
Traumatic brain injury	117 (7)	4 (5)	113 (7)
Aqueductal stenosis	104 (6)	10 (11)	94 (6)
Spontaneous hemorrhage	92 (5)	2 (2)	90 (6)
Posterior fossa cyst	63 (4)	5 (6)	58 (4)
Other intracranial cyst	80 (5)	5 (6)	75 (5)
Post-Infectious	47 (3)	4 (5)	43 (3)
Craniosynostosis	29 (2)	0 (0)	29 (0)
Other	84 (5)	5 (6)	79 (5)
**Medical history**, *n* (%)			
Inpatient antibiotic use within 12 months	399 (23)	22 (25)	377 (23)
Prior bacteremia	92 (7)	7 (11)	85 (7)
**Surgical history**, *n* (%)			
Prior CNS Surgery	300 (17)	17 (19)	283 (17)
Prior Non-CNS Surgery	283 (16)	15 (17)	268 (16)
Gastrostomy	72 (4)	6 (7)	66 (4)
Prior CSF^d^ leak	68 (4)	2 (2)	66 (4)
Tracheostomy	21 (1)	0 (0)	21 (1)
**Insurance type**, *n* (%)			
Private	974 (57)	48 (54)	926 (57)
Public	719 (42)	41 (46)	678 (42)
Self-Pay	5 (< 1)	0 (0)	5 (< 1)
Other	21 (1)	0 (0)	21 (1)
**Hospital**, *n* (%)			
A	160 (9)	4 (5)	156 (10)
B	179 (10)	7 (8)	172 (11)
C	470 (27)	28 (31)	442 (27)
D	278 (16)	20 (22)	258 (16)
E	289 (17)	10 (11)	279 (17)
F	347 (20)	20 (22)	327 (20)

The following lists the number of missing values per variable at the patient level: race (32), ethnicity (325), birth weight (791), gestational age (657), hydrocephalus etiology (1), prior bacteremia (437), prior CSF leak (73), insurance type (4)

*Abbreviations* a) central nervous system, b) intraventricular hemorrhage, c) congenital communicating hydrocephalus, d) cerebrospinal fluid

*p<0.05, **p<0.01, ***p<0.001

There were 138 shunt infections identified within 6 months of the antecedent surgery. Table 1. provides a bivariate analysis between patient level characteristics and CSF shunt infections. The only patient-level factor that differed between children who developed CSF shunt infection and those who did not was etiology of hydrocephalus. Table 2 provides a bivariate analysis between procedure-level characteristics and CSF shunt infections. There were significant differences between procedures with and without infection in procedure type, age at surgery, weight at surgery, year of shunt surgery, and use of antibiotic impregnated sutures. There was a larger proportion of infections following initial placements compared to following shunt revisions. Patients with shunt infections tended to be younger (2.25 ± 4.61 years vs 3.03 ± 4.61 years) and lower weight at surgery (13.41 ± 18.24 kg vs 15.45 ± 17.29 kg). Antibiotic impregnated sutures were associated with infections.

**Table 2:** Procedure-level characteristics for the overall cohort and in association with CSF shunt infection within 6 months

Variable	Total procedures (*n* = 3,094)	Infection present (*n* = 138)	Infection absent (*n* = 2,956)
Procedure type^*^, *n* (%)			
Initial placement	1723 (56)	89 (64)	1634 (55)
Revision	1371 (44)	49 (36)	1322 (45)
Age at surgery^**^, years, mean (SD)	3.00 (4.67)	2.25 (4.61)	3.03 (4.67)
Weight at surgery^**^, kg, mean (SD)	15.35 (17.34)	13.41 (18.24)	15.45 (17.29)
**Shunt revision reason**^**a**^, *n* (%)			
Shunt obstruction	847 (62)	25 (51)	822 (62)
Additional shunt required	117 (9)	5 (10)	112 (9)
Shunt disconnection	86 (6)	4 (8)	82 (6)
Underdrainage	61 (5)	3 (6)	58 (4)
Shunt misplacement	59 (4)	1 (2)	58 (4)
Negative exploration	36 (3)	1 (2)	35 (3)
Associated surgery requiring shunt manipulation	34 (3)	3 (6)	31 (2)
Shunt migration/outgrown distal catheter	32 (2)	2 (4)	30 (2)
Overdrainage	23 (2)	1 (2)	22 (2)
Failed ETV	5 (< 1)	0 (0)	5 (< 1)
Other	68 (5)	4 (8)	64 (5)
Procedure year*, *n* (%)			
2007	376 (12)	17 (12)	359 (12)
2008	476 (15)	20 (14)	456 (15)
2009	493 (16)	14 (10)	479 (16)
2010	464 (15)	30 (22)	434 (15)
2011	468 (15)	30 (22)	438 (15)
2012	457 (15)	16 (12)	441 (15)
2013	125 (4)	7 (5)	118 (4)
2014	128 (4)	2 (1)	126 (4)
2015	107 (4)	2 (1)	105 (4)
**Case urgency**, *n* (%)			
Elective	2109 (69)	95 (70)	2014 (69)
Add-On	433 (14)	20 (15)	413 (14)
Emergent	522 (17)	21 (15)	501 (17)
**Location of proximal catheter**, *n* (%)			
Ventricular	2744 (94)	124 (92)	2620 (94)
Subdural	101 (3)	3 (2)	98 (4)
Cyst	65 (2)	7 (5)	58 (2)
Lumbar	15 (1)	1 (1)	14 (1)
Fourth ventricle	5 (< 1)	0 (0)	5 (< 1)
**Location of distal catheter**, *n* (%)			
Peritoneal	2850 (97)	131 (98)	2719 (97)
Atrial	53 (2)	3 (2)	50 (2)
Pleural	14 (1)	0 (0)	14 (1)
Other	12 (< 1)	0 (0)	12 (< 1)
**Location of AIC**^**b**^, *n* (%)			
Proximal	248 (30)	7 (23)	241 (30)
Distal	208 (25)	9 (30)	199 (25)
Both	372 (45)	14 (47)	358 (45)
**Total surgical time,** minutes, mean (sd)	61.84 (59.60)	54.21 (32.80)	62.19 (60.53)
**Number of people in the operating room**, median (IQR^c^)	7 (6,8)	7 (6,8)	7 (6,8)
**Number of people scrubbed,** median (IQR)	4 (3,5)	4 (3,5)	4 (3,5)
Use of antibiotic impregnated sutures^**^, *n* (%)	286 (10)	22 (16)	264 (9)
**Use of intraoperative ultrasound**, *n* (%)	224 (7)	13 (9)	211 (7)
**Use of stereotactic navigation**, *n* (%)	343 (11)	8 (6)	335 (11)
**Use of intraoperative endoscope**, *n* (%)	459 (15)	14 (10)	445 (15)

The following lists the number of missing values per variable at the procedure level: case urgency (30), location of proximal catheter (159), location of distal catheter (160), total surgical time (4), number of people in operating room (177), number of people scrubbed (176), antibiotic sutures (125), intraoperative ultrasound (2), stereotactic navigation (1), intraoperative endoscope (2)

^*^P < 0.05, ^**^p < 0.01, ^***^p < 0.001

^a^Variable out of a total of 1,371 revision procedures

^b^Variable out of a total of 828 surgeries that utilized AICs

^c^Interquartile range

**Fig. 1:**
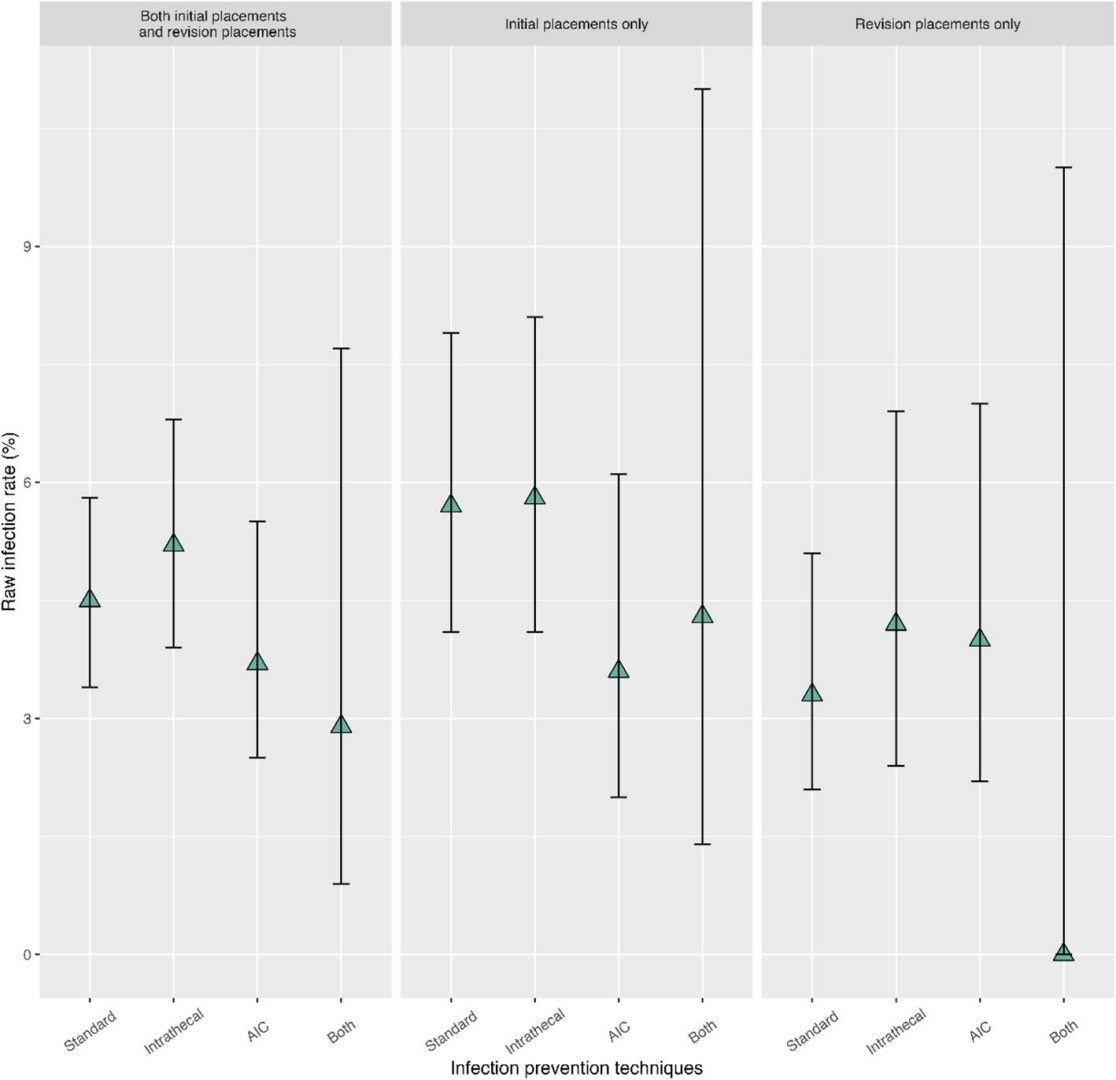
Observed rate of 6 month risk of infection (%, 95% confidence intervals) by infection prevention technique

We compared infection rates between the three shunt prevention techniques (Table 3). The overall 6-month infection rate of shunt placements (both initial and revision) was 4.5% [95% CI: 3.8,5.3], with no significant differences observed between infection prevention techniques (Fig. 1). Adjusted odds ratios generated from CBPS are also presented in Table 3. Among all procedures, compared to standard care, IT antibiotics had an aOR of 1.4, [95% CI: 0.7, 2.7], *p* = 0.4 and AICs had an aOR of 0.7, [95% CI: 0.5, 1.2], *p* = 0.2. None of the shunt infection prevention techniques showed a significant independent association with infection at 6 month when separated by initial versus revision placements.

**Table 3:** 6 month risk of infection overall and by infection prevention technique and propensity score adjusted odds ratios (aOR) by infection prevention technique

	**Infection within 6 months**			
	Raw infection rate		aOR (95% CI)	*P*-value^1^
	*n*/*N* (%)	95% CI		
**Both initial placements and revision placements**				
Standard	58/1,297 (4.5%)		Ref	
Intrathecal	50/964 (5.2%)		1.3 (0.7, 2.5)	0.48
AIC	26/695 (3.7%)		0.7 (0.5, 1.2)	0.19
Both	4/138 (2.9%)		0.8 (0.2, 2.8)	0.68
**Initial shunt placements only**				
Standard	36/634 (5.7%)		Ref	
Intrathecal	35/603 (5.8%)		1.2 (0.5, 2.9)	0.69
AIC	14/392 (3.6%)		0.6 (0.3, 1.1)	0.11
Both	4/94 (4.3%)		1.9 (0.5, 7.6)	0.39
**Revisions only**				
Standard	22/663 (3.3%)		Ref	
Intrathecal	15/361 (4.2%)		1.4 (0.5, 3.9)	0.54
AIC	12/303 (4.0%)		1.0 (0.5, 2.0)	0.93
Both	0/44 (0%)		0 (NA^2^)	NA^2^

^1^*P*-values were based on logistic regression models with inverse probability weighting, in which weights were derived from covariates balancing propensity scores

^2^*P*-value and 95% CI could not be calculated due to zero event

Table 4 reports secondary outcomes and post-operative complications within seven days of surgery. There was no significant difference in hospital length of stay between the infection prevention techniques. There were no significant differences in any other complication rates amongst the procedures except for the presence of a post-operative pseudomeningocele (1.5% in standard care group compared to 0.1% for both IT antibiotics and AICs) and other complications (11% for both IT antibiotics and AICs compared to other groups).

**Table 4:** Secondary outcomes by infection prevention technique

		Infection Prevention Technique			
	Overall (*N* = 3094)	Standard (*N* = 1297)	IT Abx (*N* = 964)	AIC (*N* = 695)	Both (*N* = 138)
**Length of stay** in days, mean (SD)	14.1 (31.3)	12.8 (31.7)	15.2 (30.8)	14.9 (32.2)	13.7 (25.5)
**Post-op complication**, rate (%)					
Bacteremia	12/3,088 (0.4%)	5/1,293 (0.4%)	3/963 (0.3%)	3/694 (0.4%)	1/138 (0.7%)
CSF leak	34/3,088 (1.1%)	15/1,293 (1.2%)	12/963 (1.2%)	5/694 (0.7%)	2/138 (1.4%)
Pseudomeningocele^***^	23/3,087 (0.7%)	19/1,293 (1.5%)	1/963 (0.1%)	1/693 (0.1%)	2/138 (1.4%)
Wound breakdown	8/3,088 (0.3%)	2/1,293 (0.2%)	3/963 (0.3%)	1/694 (0.1%)	2/138 (1.4%)
Abx treatment for wound site	10/3,087 (0.3%)	6/1,292 (0.5%)	2/963 (0.2%)	1/694 (0.1%)	1/138 (0.7%)
Meningitis	24/3,086 (0.8%)	15/1,293 (1.2%)	7/963 (0.7%)	2/693 (0.3%)	0/137 (0%)
Bowel perforation	0/3,088 (0%)	0/1,293 (0%)	0/963 (0%)	0/694 (0%)	0/138 (0%)
Other complications^***^	149/3,088 (4.8%)	46/1,292 (3.6%)	58/964 (6.0%)	30/694 (4.3%)	15/138 (11%)

^*^P < 0.05, ^**^p < 0.01, ^***^p < 0.001

## Discussion

We took advantage of the natural experiment that occurred in PHIS + hospitals from 2007 to 2012 with the use of IT antibiotics and AIC to compare these techniques to standard care in the cohort of children undergoing initial CSF shunt placement and CSF shunt revisions. In this retrospective analysis of over 3,000 low-risk surgeries at six institutions between 2007 through 2015, there were no differences in 6-month infection rates between standard care, IT antibiotics and AICs. AICs tended to have a favorable odds ratio compared to standard care and IT antibiotics tended to have an unfavorable odds ratio compared to standard care; however, no significant differences were observed between the techniques. This was observed both when evaluating all procedures combined and then both initial and revision placements separately.

The HCRN has implemented multiple peri-operative infection prevention protocols over the last 15 years. In 2007, the HCRN protocol recommended that surgeons utilize a one-time instillation of IT antibiotics, consisting of vancomycin and gentamicin, for all shunt surgeries in addition to pre-operative intravenous antibiotics [16]. A 2011 study demonstrated a reduction in infection rates from 8.8% to 5.7% (*p* = 0.003) following the implementation of the IT antibiotic protocol [16]. Subsequently there was increasing adoption and research into the efficacy and utility of AICs [33–42] coated with rifampin and clindamycin [43]. The subsequent HCRN protocol replaced the use of IT antibiotics with AICs [17]. A subsequent 2016 study showed a similar infection rate of 6.0% (*p* = 0.002) following the protocol replacing IT antibiotics with AIC [17]. Our recent study reviewed utilization trends of the three infection prevention techniques in six PHIS + hospitals and demonstrated that AIC use increased and IT antibiotic use decreased during the study period, except for Hospital B which consistently used AICs [20].

Our unadjusted 6 month infection rates across all techniques were 4.5% for standard care, 5.2% for IT antibiotics, and 3.7% for AICs. These rates are lower than the HCRN cohorts. Most previous studies evaluating IT antibiotics and AICs incorporated all children who received a shunt surgery prior to enrollment, which included children presenting with a previous shunt infection. Our cohort design allowed us to have complete shunt history and thus minimize variation in infection risk. Hence lower infection rates were observed due to the inherently lower risk patient population within our study.

A 2012 study by Simon et al. evaluated 1000 children undergoing shunt placements, and after controlling for baseline factors, it was noted that infection risk was most significantly associated with the need for revision [11]. In this and multiple other studies, it was concluded that relatively few patient, medical, or surgical risk factors – other than revision surgery itself—were associated with first infection [10, 11, 26, 44] Paradoxically, our cohort demonstrates a higher percentage of infections in the initial placement compared to the revision placements. Of note, we observed a decrease in the number of overall infections following the year 2012. This is explained by the fact that no new children were enrolled in the subject pool following this year, but infection events were continued to be monitored for the existing study population.

Since this is a retrospective cohort study, we measured the association between techniques and infection risk, rather than causality. It might be argued that a clinical trial is optimal, however, the use of a large database permits us to efficiently capitalize upon the existence of detailed data on large numbers of CSF shunt surgeries (far larger cohorts than previously assembled) and allowed us to examine a wider spectrum of children. We were also able to use sophisticated analytic approaches to optimize predicted probability of treatment given practice variation we observed in earlier work [20]. This study provides relevant information about the newest CSF shunt infection prevention technique in use today, AIC, and suggests limited benefit in a low-risk population. There is a relatively small number of surgeons and hospitals that limit our ability to study surgeon and hospital effects on patient outcome systematically; however, the multi-institutional nature of this study gives it greater generalizability than previous studies. Similarly, while minority children are under-represented in these data, its multi-institutional nature provides greater generalizability than previous studies. Because we observed much lower infections across all three techniques than previously reported, using standard care as the reference group, our sample sizes provided only 12% power to detect the difference between intrathecal vs. standard, and 13% power to detect the difference between AIC versus standard care. Therefore, because of the relatively rare occurrence of infection events, even with our large multi-year sample we were under-powered for most comparisons. Despite this limitation, this real-world evidence provides little support for routine use of IT antibiotics or AICs compared to standard care in low-risk CSF shunt surgeries.

## Conclusion

We did not observe a difference in 6 month infection rates or adjusted odds of infection between AIC or IT compared to standard care for children undergoing initial CSF shunt placement and CSF shunt revisions. Compared to previous studies, the benefit provided by AICs and IT compared to standard care may not be as large as previously believed amongst low-risk patients once cohorts are appropriately balanced. The real-world benefit of AIC among low-risk patients should be evaluated carefully using current data given interim changes in surgical practice and their widespread adoption.

## Data Availability

The datasets used and/or analyzed during the current study are available from the corresponding author upon reasonable request.

## Abbreviations

AIC: Antibiotic Impregnated Catheter
CSF: Cerebrospinal fluid
CI: Confidence interval
CBPS: Covariate balancing propensity score
HCRN: Hydrocephalus Clinical Research Network
IT: Intrathecal

## References

[1] Williams MA, McAllister JP, Walker ML, Kranz DA, Bergsneider M, Del Bigio MR (2007). Priorities for hydrocephalus research: report from a National Institutes of Health-sponsored workshop. J Neurosurg.

[2] Fletcher JM, Bohan TP, Brandt ME, Kramer LA, Brookshire BL, Thorstad K (1996). Morphometric evaluation of the hydrocephalic brain: relationships with cognitive development. Childs Nerv Syst.

[3] Dewan MC, Rattani A, Mekary R, Glancz LJ, Yunusa I, Baticulon RE, et al. Global hydrocephalus epidemiology and incidence: systematic review and meta-analysis. J Neurosurg. 2018;130(4):1065–79. 10.3171/2017.10.JNS17439. 10.3171/2017.10.JNS17439 29701543

[4] Whitehead WE, Weiner HL (2022). Infantile and childhood hydrocephalus. N Engl J Med.

[5] Kestle JR (2003). Pediatric hydrocephalus: current management. Neurol Clin.

[6] Browd SR, Ragel BT, Gottfried ON, Kestle JR (2006). Failure of cerebrospinal fluid shunts: part I: obstruction and mechanical failure. Pediatr Neurol.

[7] Browd SR, Gottfried ON, Ragel BT, Kestle JR (2006). Failure of cerebrospinal fluid shunts: part II: overdrainage, loculation, and abdominal complications. Pediatr Neurol.

[8] Kestle J, Drake J, Milner R, Sainte-Rose C, Cinalli G, Boop F (2000). Long-term follow-up data from the shunt design trial. Pediatr Neurosurg.

[9] Simon TD, Riva-Cambrin J, Srivastava R, Bratton SL, Dean JM, Kestle JR (2008). Hospital care for children with hydrocephalus in the United States: utilization, charges, comorbidities, and deaths. J Neurosurg Pediatr.

[10] Simon TD, Whitlock KB, Riva-Cambrin J, Kestle JR, Rosenfeld M, Dean JM (2012). Revision surgeries are associated with significant increased risk of subsequent cerebrospinal fluid shunt infection. Pediatr Infect Dis J.

[11] Simon TD, Butler J, Whitlock KB, Browd SR, Holubkov R, Kestle JR (2014). Risk factors for first cerebrospinal fluid shunt infection: findings from a multi-center prospective cohort study. J Pediatr.

[12] Vinchon M, Dhellemmes P (2006). Cerebrospinal fluid shunt infection: risk factors and long-term follow-up. Childs Nerv Syst.

[13] Kulkarni A, Cochrane D, McNeely PD, Shams I (2007). Medical, social, and economic factors associated with health-related quality of life in Canadian children with hydrocephalus. J Neurosurg.

[14] Wong JM, Ziewacz JE, Ho AL, Panchmatia JR, Bader AM, Garton HJ (2012). Patterns in neurosurgical adverse events: cerebrospinal fluid shunt surgery. Neurosurg Focus.

[15] Mallucci CL, Jenkinson MD, Conroy EJ, Hartley JC, Brown M, Dalton J (2019). Antibiotic or silver versus standard ventriculoperitoneal shunts (BASICS): a multicentre, single-blinded, randomised trial and economic evaluation. Lancet.

[16] Kestle JR, Riva-Cambrin J, Wellons JC, Kulkarni AV, Whitehead WE, Walker ML (2011). A standardized protocol to reduce cerebrospinal fluid shunt infection: the Hydrocephalus Clinical Research Network Quality Improvement Initiative. J Neurosurg Pediatr.

[17] Kestle JR, Holubkov R, Douglas Cochrane D, Kulkarni AV, Limbrick DD, Luerssen TG (2016). A new Hydrocephalus Clinical Research Network protocol to reduce cerebrospinal fluid shunt infection. J Neurosurg Pediatr.

[18] Qiu Y, Wu Y (2020). Efficacy of antibiotic-impregnated shunt versus conventional shunts to reduce cerebrospinal fluid infections in children: a systematic review and meta-analysis. Exp Ther Med.

[19] Tamber MS, Jensen H, Clawson J, Nunn N, Wellons JC, Smith J (2024). Shunt infection prevention practices in Hydrocephalus Clinical Research Network-Quality: a new quality improvement network for hydrocephalus management. J Neurosurg Pediatr.

[20] Podkovik S, Zhou C, Coffin SE, Hall M, Hauptman JS, Kronman MP, et al. Utilization trends in cerebrospinal fluid shunt infection prevention techniques in the United States from 2007 to 2015. J Neurosurg Pediatr. 2024;33(4):349–58. 10.3171/2023.11.PEDS2337. 10.3171/2023.11.PEDS2337 PMC10810681 38181501

[21] Harris PA, Taylor R, Thielke R, Payne J, Gonzalez N, Conde JG (2009). Research electronic data capture (REDCap)–a metadata-driven methodology and workflow process for providing translational research informatics support. J Biomed Inform.

[22] Harris PA, Taylor R, Minor BL, Elliott V, Fernandez M, O'Neal L (2019). The REDCap consortium: building an international community of software platform partners. J Biomed Inform.

[23] Rosenbaum PR, Rubin DB (1983). The central role of the propensity score in observational studies for causal effects. Biometrika.

[24] Imai K, Ratkovic M (2014). Covariate balancing propensity score. J R Stat Soc.

[25] Simon TD, Hall M, Dean JM, Kestle JR, Riva-Cambrin J (2010). Reinfection following initial cerebrospinal fluid shunt infection. J Neurosurg Pediatr.

[26] Simon TD, Hall M, Riva-Cambrin J, Albert JE, Jeffries HE, Lafleur B (2009). Infection rates following initial cerebrospinal fluid shunt placement across pediatric hospitals in the United States. Clinical article. J Neurosurg Pediatr.

[27] Simon TD, Mayer-Hamblett N, Whitlock KB, Langley M, Kestle JR, Riva-Cambrin J (2014). Few patient, treatment, and diagnostic or microbiological factors, except complications and intermittent negative Cerebrospinal Fluid (CSF) cultures during first CSF shunt infection, are associated with reinfection. J Pediatric Infect Dis Soc.

[28] Simon TD, Whitlock KB, Riva-Cambrin J, Kestle JR, Rosenfeld M, Dean JM (2012). Association of intraventricular hemorrhage secondary to prematurity with cerebrospinal fluid shunt surgery in the first year following initial shunt placement. J Neurosurg Pediatr.

[29] Fong C, Ratkovic M, Kosuke I, et al. CBPS: Covariate balancing propensity score. Published online January 18, 2022. https://CRAN.R-project.org/package=CBPS.

[30] Greifer N. WeightIt: Weighting for covariate balance in observational studies. Published online June 28, 2022. https://CRAN.R-project.org/package=WeightIt.

[31] R Core Team 2022. R: A Language and environment for statistical computing. https://www.r-project.org/.

[32] Greifer N. cobalt: Covariate balance tables and plots. Published online November 3, 2021. https://CRAN.Rproject.org/package=cobalt.

[33] James G, Hartley JC, Morgan RD, Ternier J (2014). Effect of introduction of antibiotic-impregnated shunt catheters on cerebrospinal fluid shunt infection in children: a large single-center retrospective study. J Neurosurg Pediatr.

[34] Klimo P, Thompson CJ, Baird LC, Flannery AM, Pediatric Hydrocephalus Systematic R, Evidence-Based Guidelines Task F (2014). Pediatric hydrocephalus: systematic literature review and evidence-based guidelines. Part 7: Antibiotic-impregnated shunt systems versus conventional shunts in children: a systematic review and meta-analysis. J Neurosurg Pediatr.

[35] Sciubba DM, Stuart RM, McGirt MJ, Woodworth GF, Samdani A, Carson B (2005). Effect of antibiotic-impregnated shunt catheters in decreasing the incidence of shunt infection in the treatment of hydrocephalus. J Neurosurg.

[36] Govender ST, Nathoo N, van Dellen JR (2003). Evaluation of an antibiotic-impregnated shunt system for the treatment of hydrocephalus. J Neurosurg.

[37] Kan P, Kestle J (2007). Lack of efficacy of antibiotic-impregnated shunt systems in preventing shunt infections in children. Childs Nerv Syst..

[38] Eymann R, Chehab S, Strowitzki M, Steudel WI, Kiefer M (2008). Clinical and economic consequences of antibiotic-impregnated cerebrospinal fluid shunt catheters. J Neurosurg Pediatr.

[39] Hayhurst C, Cooke R, Williams D, Kandasamy J, O'Brien DF, Mallucci CL (2008). The impact of antibiotic-impregnated catheters on shunt infection in children and neonates. Childs Nerv Syst.

[40] Kandasamy J, Dwan K, Hartley JC, Jenkinson MD, Hayhurst C, Gatscher S (2011). Antibiotic-impregnated ventriculoperitoneal shunts–a multi-centre British paediatric neurosurgery group (BPNG) study using historical controls. Childs Nerv Syst.

[41] Parker SL, Anderson WN, Lilienfeld S, Megerian JT, McGirt MJ (2011). Cerebrospinal shunt infection in patients receiving antibiotic-impregnated versus standard shunts. J Neurosurg Pediatr.

[42] Aryan HE, Meltzer HS, Park MS, Bennett RL, Jandial R, Levy ML (2005). Initial experience with antibiotic-impregnated silicone catheters for shunting of cerebrospinal fluid in children. Childs Nerv Syst.

[43] Jenkinson MD, Gamble C, Hartley JC, Hickey H, Hughes D, Blundell M (2014). The British antibiotic and silver-impregnated catheters for ventriculoperitoneal shunts multi-centre randomised controlled trial (the BASICS trial): study protocol. Trials.

[44] McGirt MJ, Zaas A, Fuchs HE, George TM, Kaye K, Sexton DJ (2003). Risk factors for pediatric ventriculoperitoneal shunt infection and predictors of infectious pathogens. Clin Infect Dis.

